# Regression of Fibrosis and Reversal of Cirrhosis in Rats by Galectin Inhibitors in Thioacetamide-Induced Liver Disease

**DOI:** 10.1371/journal.pone.0075361

**Published:** 2013-10-09

**Authors:** Peter G. Traber, Hsin Chou, Eliezer Zomer, Feng Hong, Anatole Klyosov, Maria-Isabel Fiel, Scott L. Friedman

**Affiliations:** 1 Galectin Therapeutics Inc, Norcross, Georgia, United States of America; 2 Department of Medicine, Emory University School of Medicine, Atlanta, Georgia, United States of America; 3 Division of Liver Diseases, Icahn School of Medicine at Mount Sinai, New York City, New York, United States of America; 4 Department of Pathology, Icahn School of Medicine at Mount Sinai, New York City, New York, United States of America; University of Navarra School of Medicine and Center for Applied Medical Research (CIMA), Spain

## Abstract

Galectin-3 protein is critical to the development of liver fibrosis because galectin-3 null mice have attenuated fibrosis after liver injury. Therefore, we examined the ability of novel complex carbohydrate galectin inhibitors to treat toxin-induced fibrosis and cirrhosis. Fibrosis was induced in rats by intraperitoneal injections with thioacetamide (TAA) and groups were treated with vehicle, GR-MD-02 (galactoarabino-rhamnogalaturonan) or GM-CT-01 (galactomannan). In initial experiments, 4 weeks of treatment with GR-MD-02 following completion of 8 weeks of TAA significantly reduced collagen content by almost 50% based on Sirius red staining. Rats were then exposed to more intense and longer TAA treatment, which included either GR-MD-02 or GM-CT-01 during weeks 8 through 11. TAA rats treated with vehicle developed extensive fibrosis and pathological stage 6 Ishak fibrosis, or cirrhosis. Treatment with either GR-MD-02 (90 mg/kg ip) or GM-CT-01 (180 mg/kg ip) given once weekly during weeks 8–11 led to marked reduction in fibrosis with reduction in portal and septal galectin-3 positive macrophages and reduction in portal pressure. Vehicle-treated animals had cirrhosis whereas in the treated animals the fibrosis stage was significantly reduced, with evidence of resolved or resolving cirrhosis and reduced portal inflammation and ballooning. In this model of toxin-induced liver fibrosis, treatment with two galectin protein inhibitors with different chemical compositions significantly reduced fibrosis, reversed cirrhosis, reduced galectin-3 expressing portal and septal macrophages, and reduced portal pressure. These findings suggest a potential role of these drugs in human liver fibrosis and cirrhosis.

## Introduction

Liver fibrosis results from a range of chronic inflammatory diseases including viral hepatitis, alcoholic and non-alcoholic steatohepatitis, immune injury, primary biliary cirrhosis, and others [1]. The accumulation of collagen following chronic inflammation is driven by a cascade of events that involves cytokines produced by both liver resident cells and circulating immune cells. As a result of these inflammatory stimuli, quiescent stellate cells in the space of Disse are activated to myofibroblast-like cells to secrete collagen. The accumulation of collagen and other extracellular matrix molecules far exceeds their degradation by metalloproteases released from resident and infiltrating macrophages. With ongoing injury, fibrosis develops initially around either portal tracts or central veins, eventually forming bridging fibrosis with nodule formation surrounded by thick bands of fibrous tissue, culminating in cirrhosis. The distorted architecture of the cirrhotic liver leads to complications of portal hypertension, reduced hepatocellular function, and a risk of hepatocellular carcinoma.

While therapies for the underlying diseases leading to fibrosis have advanced, for example those for viral hepatitis, there are currently no approved therapies for treatment of fibrosis. Many potential anti-fibrotic targets have been identified and a number of drugs have been tested in clinical trials [2], [3].

One recently described potential target for therapy is the galectin-3 protein. Galectins are a family of 15 proteins that have a carbohydrate binding domain that binds to terminal galactose residues on macromolecules such as glycoproteins [4], [5]. Galectin-3 protein, a prominent galectin expressed in immune cells and markedly increased in inflammation [5]–[7], has recently been implicated in the pathogenesis of fibrosis in several disease models. For example, galectin-3 null mice are resistant to developing liver fibrosis due to carbon tetrachloride [8], and to the development of steatohepatitis and fibrosis when fed a high fat diet [9]. Moreover, other organs in galectin-3 null mice are resistant to fibrogenesis including lung [10] and kidney [11]. Based on these data, it appears that galectin-3 protein is implicated in the development of fibrosis resulting from inflammatory or toxic insults, thereby establishing a rationale to antagonize its function to treat fibrosis.

In this study we have evaluated the effect of complex carbohydrate drugs that bind to galectin-3 protein, as well as galectin-1, using a model of hepatic fibrosis and cirrhosis in rats. These agents, GR-MD-02 and GM-CT-01, appear to be well tolerated and promote significant regression in fibrosis following thioacetamide-induced liver injury.

## Materials and Methods

### Drug Compounds

GM-CT-01 is a linear polysaccharide, molecular weight of approximately 54 KDa, derived from guar galactomannan that is comprised of a backbone of (1,4)-linked β-D-mannose with side molecules of (1,6)-linked β-D-galactose on average every 1.7 mannose residues. In these studies, GM-CT-01 was produced as described in US patent #7,893,252.

GR-MD-01 is a galacto-rhamnogalacturonan polysaccharide, molecular weight of approximately 120 KDa, with a backbone comprised predominantly of 1,4-linked galacturonic acid (GalA) moieties, with a lesser backbone composition of alternating 1,4-linked GalA and 1,2-linked rhamnose, which in-turn is linked to any number of side chains, including predominantly 1,4-β-D-galactose. GR-MD-01 was produced as described in US patent #8,236,780. GR-MD-02 is a galactoarabino-rhamnogalacturonan polysaccharide, molecular weight of approximately 50 KDa, which has the same backbone as GR-MD-01 with side chains that include both 1,4-β-D-galactose (Gal) and 1,5-α-L-arabinose (Ara). GR-MD-02 was produced as described in patent #PCT/US12/55311.

### Rat Fibrosis Model

Two sets of analyses were conducted, the first at Fudan University in China and the second at the Icahn School of Medicine at Mount Sinai in New York City. An experiment comparing GR-MD-01 and GR-MD-02 was performed by investigators at Fudan University (Shanghai, China) under a contract to Galectin Therapeutics using male Sprague–Dawley rats between 160 and 200 g obtained from the Animal Research Center of Fudan University which were maintained according to the Guide for the Care and Use of Laboratory Animals (Institute of Laboratory Animal Resources, 1996, Nat. Acad. Press) and approved by the Fudan University (Shanghai, China) Institutional Animal Care and Use Committee (IACUC). At the end of experiments, animals were euthanized under phenobarbital anesthesia. After an acclimation period of two weeks, an eight-week fibrosis induction period was initiated, in which all rats were subjected to intraperitoneal (IP) injections of sterile solutions of TAA (Bomei Biological and Technological Co., Product No. BM1257-1), dissolved in 0.9% saline, administered twice weekly. During the initial week of treatment, the TAA regimen was biweekly IP injection of 0.25 g/kg body weight, followed by seven weeks regimen of biweekly injection of 0.20 g/kg body weight for a total of eight injections. To assess for the progression of fibrosis, at the end of weeks 4 and 8, two rats per time point were euthanized and the liver examined histologically. During this period one rat died of abdominal hemorrhage during TAA injection.

Experiments comparing GR-MD-02 with GM-CT-01 were conducted at Mount Sinai using male Sprague–Dawley rats between 280 and 300 g (Jackson Laboratory), which were maintained according to NIH guidelines and approved by the Mount Sinai Institutional Animal Care and Use Committee (IACUC). Rats were kept in the Animal Care Facility with a 12 hour light–dark cycle at constant temperature, with free access to water during the study period. Animals were administered 150 mg/kg of TAA (Sigma Chemical Co., St. Louis, MO, USA) by IP, three times weekly for 11 weeks to induce cirrhosis. Beginning in week 8, group 1 rats were administered 0.9% NaCl intraperitoneally (IP) twice weekly for four weeks. Beginning in week 8, Groups 2, 3 and 4, rats were administered GR-MD-02 IP at concentrations of 60 mg/kg twice per week for four weeks, 60 mg/kg once per week for four weeks, and 90 mg/kg once per week for four weeks, respectively. Beginning in week 8, groups 5, 6 and 7 were administered GM-CT-01 IP at concentrations of 105 mg/kg twice per week for four weeks, 105 mg/kg once per week for four weeks, and 180 mg/kg once per week for four weeks, respectively.

At the end of the treatment period, rats were placed under anesthesia using isofluorane between 1–5% through inhalation and a laparotomy was performed. At the time of sacrifice, portal pressure was measured using a 16 G angiocatheter introduced into the portal vein to measure the height of a water column. The liver was removed, weighed, and pieces from the largest lobes were used for further analysis. The spleen was also removed and weighed before being discarded.

### Assays of Serum Aminotransferases

In the experiment comparing GR-MD-02 and GM-CT-01, blood was collected in EDTA (1.5 mg per ml of blood) containing microfuge tubes, pelleted at 5,000 rpm for 10 min, and plasma samples were obtained for measuring serum alanine and aspartate aminotransferase (ALT/AST) and creatinine levels using VITROS® 5, 1 FS (Ortho Clinical Diagnostics). If needed to ensure the assay was in the linear range, plasma was diluted with VITROS® containing 7% BSA.

### Liver Histology and Fibrosis Quantification

In the experiment to compare GR-MD-01 and GR-MD-02, livers were fixed in 10% formalin, embedded in paraffin, sectioned at 4-mm thickness, and stained with hematoxylin & eosin (H&E) and separately for Sirius red. All pathologic evaluations were made by an experienced pathologist on a random and blinded basis. Collagen surface density was quantified using a computerized image analysis system (KS400 Image Analysis Software with a ZEISS microscope). Slides were also scored by the modified Ishak scoring system (0–6) by an experienced liver pathologist who was blinded to the animal groups.

In the experiment comparing GR-MD-02 and GM-CT-01, livers were fixed in 3.7% formalin, embedded in paraffin and cut at 4-mm thickness and stained with H&E for histological examination. Liver sections were also stained with 0.1% Sirius red and 0.1% Fast Green in saturated picric acid (Sigma Chemical Co.). Four Sirius red-stained slides per animal were taken, with nine images taken randomly per slide for a total of 36 images per animal for collagen quantification using computerized BIOQUANT Life Science morphometry® system.

In addition, thirty-three H&E-stained liver sections from four treatment groups were evaluated histologically in a blind fashion. Eight histological features were scored from six random sections at 100×magnification so that a minimum of 48 scores from each slide was determined. These features were ductular reaction (score 0–3), portal and lobular inflammation independently (0- none, 1-mild, 2-moderate, 3-severe), presence or absence of atypical cells comprising the ductular reaction, degree of steatosis (0-none, 1<30%, 2>30 but <60%, 3- >60%), type of steatotic vacuoles (microvesicular or macrovesicular) and ballooning degeneration (0-none, 1-occasional, 2-more than occasional, 3-numerous cells). Additionally, presence of pigment within the parenchyma was evaluated as being present or absent. Degree of fibrosis was evaluated using Sirius red-stained slides. Both the Scheuer scheme (0–4) as well as the Ishak fibrosis scores (0–6) were rendered.

### Cell Culture Experiments

LX-2 cells [12] and primary human stellate cells, isolated as previously described [13], were maintained in Dulbecco’s Modified Eagle Medium with high glucose containing 10% fetal bovine serum and 1% Penicillin-streptomycin antibiotics (Gibco, Invitrogen). Cells were treated with GM-CT-01, GR-MD-01, or GR-MD-02 for 12, 24, 48 or 72 hours at concentrations of 0.1 mg/ml and up to 2 mg/ml in medium with 0.02% BSA or 10% fetal bovine serum.

### Cell Proliferation Assay

DNA synthesis was assayed by measuring ^3^H-thymidine incorporation. LX-2 cells were seeded at a density of 20,000 cells per well in 24-well plates. After 24 hours, the medium was changed to Dulbecco’s Modified Eagle’s medium containing 0.2% BSA for 12 hours, and the cells were then treated with of 0.1 mg/ml or 1 mg/ml of GM-CT-01, GR-MD-01, GR-MD-02 for an additional 12 or 24 hours, and 1 µCi/mL ^3^H-thymidine was added 4 hours before harvesting. Cells were then washed three times with ice-cold PBS and fixed in methanol for 30 minutes at 4°C. Cells were solubilized in 0.25% sodium hydroxide/0.25% sodium dodecyl sulfate. After neutralization with 1 N hydrochloric acid, radioactivity was measured using a scintillation counter (Beckman Coulter).

### Apoptosis Analysis

Evidence of apoptosis was examined using the Annexin V apoptosis detection kit APC (Ebioscience) followed by fluorescent activated cell sorting, as per the manufacturer’s instructions. To detect fragmented or apoptotic DNA (small DNA fragments) in LX-2 cells, DNA was isolated from LX-2 cells treated with 0.1 mg/ml of GM-CT-01, GR-MD-01 or GR-MD-02 and vehicle for 48 hours using the Apoptotic DNA Ladder Extraction Kit (BioVision, Mountain View, USA) according to the manufacturer’s guidelines. Samples were run in 2% agarose gels, stained with ethidium bromide, and visualized by transillumination with UV light.

### Western Blot

Total protein was extracted from cells or liver tissue using RIPA lysis buffer (50 mM Tris-HCl pH = 8, 150 mM NaCl, 1% IGEPAL, 0.5% sodium deoxycholate and 0.1% SDS) with complete protease inhibitor mixture and protein phosphatase inhibitor mixtures (Roche and Thermal Fisher). Protein concentration was determined with a Bio-Rad DC kit (Bio-Rad). Antibodies used for analysis of LX-2 cells were as follows: α-SMA (Millipore, #04-1094), MMP2 (Abcam, #7298), MCP-1 (Santa Cruz, Sc-130328) and GAPDH (Abcam, #9482). Antibodies used for analysis of liver tissue were as follows: rabbit anti-collagen type I (1∶2,000) (Rockland), mouse anti-α-SMA (1∶500) (Abcam), and rabbit anti-GAPDH (1∶2,000) (Santa Cruz).

### Reverse Transcription and Real-Time Quantitative PCR

In the TAA-rat experiment comparing GR-MD-02 and GM-CT-01, and in the LX-2 cell experiments, mRNA from 100 mg of rat liver tissues or cells was extracted and purified using an RNeasy Mini kit (Qiagen, Valencia, CA, USA), and 1 µg of total mRNA was reverse transcribed into complementary DNA (cDNA) using Sprint™ RT Complete-RNA to cDNA EcoDryTM Premix (Double Primed) tubes (Clontech, Mountain View, CA) and mRNA for various markers was analyzed by quantitative PCR using iQ SYBR Green Supermix (Bio-Rad) on the LightCycler® 480 Real-Time PCR System (Roche). Data were represented as the relative expression of fibrogenic genes after normalizing to GAPDH.

### Zymography

MMP-2 enzymatic activity in LX-2 was determined using Novex 10% Zymogram gelatin gel (Invitrogen) following Invitrogen protocols.

### Galectin-3 Immunohistochemistry

Slides were de-paraffinized using xylene and antigen retrieval was performed using Diva De-Cloaker (Biocare, lot #111612) at 120°C for 30 seconds, then 95°C for 15 seconds. Slides were rinsed twice with TBS Auto-Wash (Biocare, lot #03813B) and Sniper (Biocare, lot #072312) was applied as a protein block for 60 minutes. The primary antibody for galectin-3 (anti-galectin-3 rabbit polyclonal from PeproTech, cat. #500-p246) was serially diluted to the working concentration of 0.1 µg/mg, applied and left on the slides overnight at 4°C. Slides were rinsed twice with TBS Auto-Wash, peroxidaze 1 (Biocare, lot #041012) as a peroxidase blocker was applied for 5 minutes, and then rinsed twice with TBS Auto-Wash. The secondary antibody (biotinylated goat anti-rabbit IgG from Jackson ImmunoResearch Laboratories, cat #H10061) was diluted 1∶500 from the original concentration of 1.3 mg/mg with DaVinci Green antibody diluent (Biocare, lot #110612) and applied for 30 min. Slides were rinsed twice with TBS Auto-Wash, and the tertiary antibody (ABC Elite; Vector Labs) was applied for 30 minutes and, after two washes, were developed with chromagen (Sigma Fast DAB, Sigma lot #LSBB5930) for 5 minutes. Slides were counterstained with hematoxylin.

## Results

### Evaluation of Anti-fibrotic Activity of Two Galacto-rhamnoglucuronate Compounds

**Figure 1:**
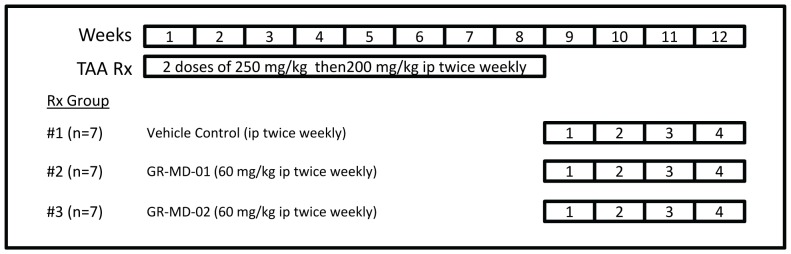
Experimental design. TAA = thioacetamide; ip = intraperitoneal injection.

Initially, GR-MD-01 and GR-MD-02 were evaluated in TAA-treated rats to choose the best agent to take forward in future experiments. Rats were treated with TAA for eight weeks, for a cumulative amount of administered TAA of 3200 mg/kg, followed by four weeks of treatment with GR-MD-01 or GR-MD-02 at doses of 60 mg/kg twice weekly or 0.9% saline as a vehicle control (Figure 1). The animals tolerated the treatments well with no obvious adverse effects observed.

**Figure 2:**
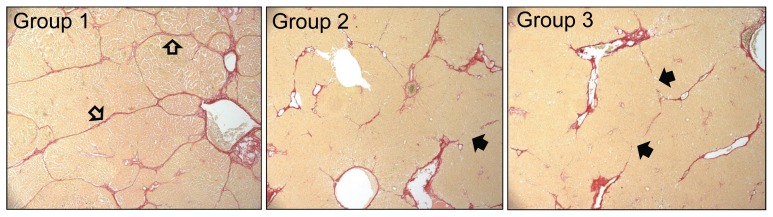
Representative histology of Sirius red stained liver sections from experiment described in **Figure 1**. A photomicrograph was chosen from each experimental group that was approximately equal to the mean of the group for percent area stained with Sirius red. Open arrows = strands of bridging fibrosis; Closed arrows = incomplete (broken) strands of bridging fibrosis.

Figure 2 shows representative histological pictures stained with Sirius red of liver sections from each group. Livers from vehicle-treated animals had collagen in both portal and central regions with well-formed strands of collagen bridging the portal and central areas (open arrows). The treated groups (groups 2 and 3) had reduced amounts of collagen, and significantly, had many fewer areas of bridging fibrosis, with evidence of incomplete bridging suggesting resolution of fibrosis (solid arrows).

**Figure 3:**
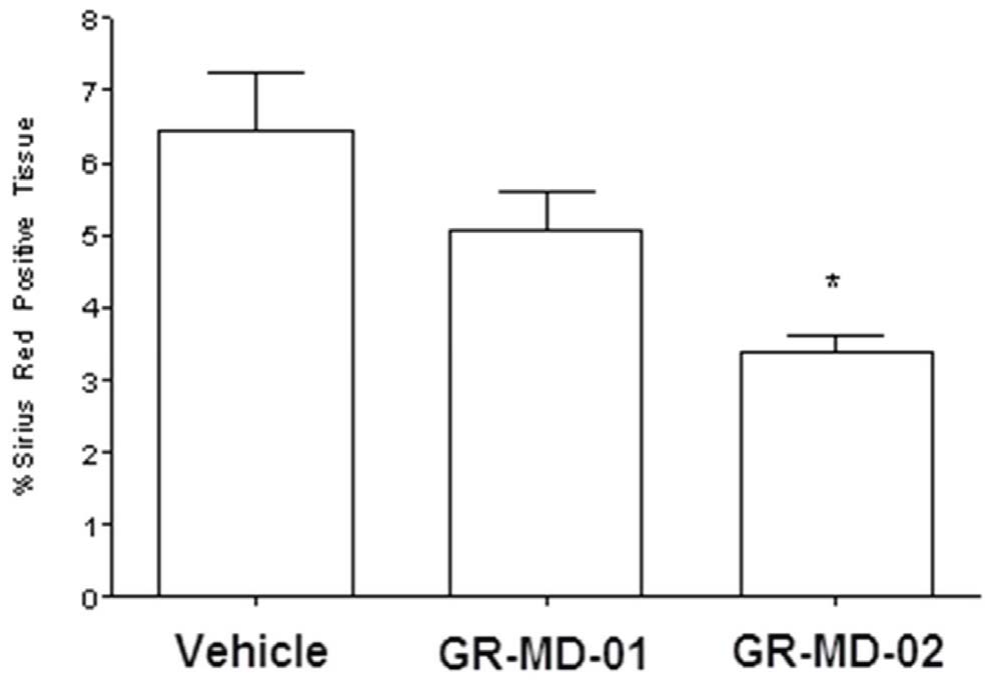
Graphical representation of the percentage Sirius red positive tissue from experiment described in **Figure 1**. Statistical analysis performed was One Way ANOVA with a Bonferroni’s post-test comparing the three groups. * = p<0.05 compared to Vehicle Group.

The amount of collagen between groups was evaluated blindly using digital morphometric quantification of Sirius red staining of multiple slides from each animal, as described in Methods (Figure 3). The vehicle-treated control animals after four weeks of TAA treatment had significant fibrosis comprising between 6% and 7% of the total area of liver based on Sirius red staining and morphometry. GR-MD-01 at a dose of 60 mg/kg twice weekly reduced the amount of collagen, but the difference from vehicle control did not reach significance. GR-MD-02 at a dose of 60 mg/kg twice weekly resulted in a marked and statistically significant reduction in Sirius red stained area.

The results of this experiment demonstrate that both compounds had an effect on reducing fibrosis in TAA-treated rats. Moreover, in addition to a reduction in collagen there were architectural changes suggesting regression of the severity of fibrosis with fewer fibrotic bridges. Because GR-MD-02 had a more robust effect further experiments used this agent.

### Evaluation of Anti-fibrotic Activity of GR-MD-2 and GM-CT-01

**Figure 4:**
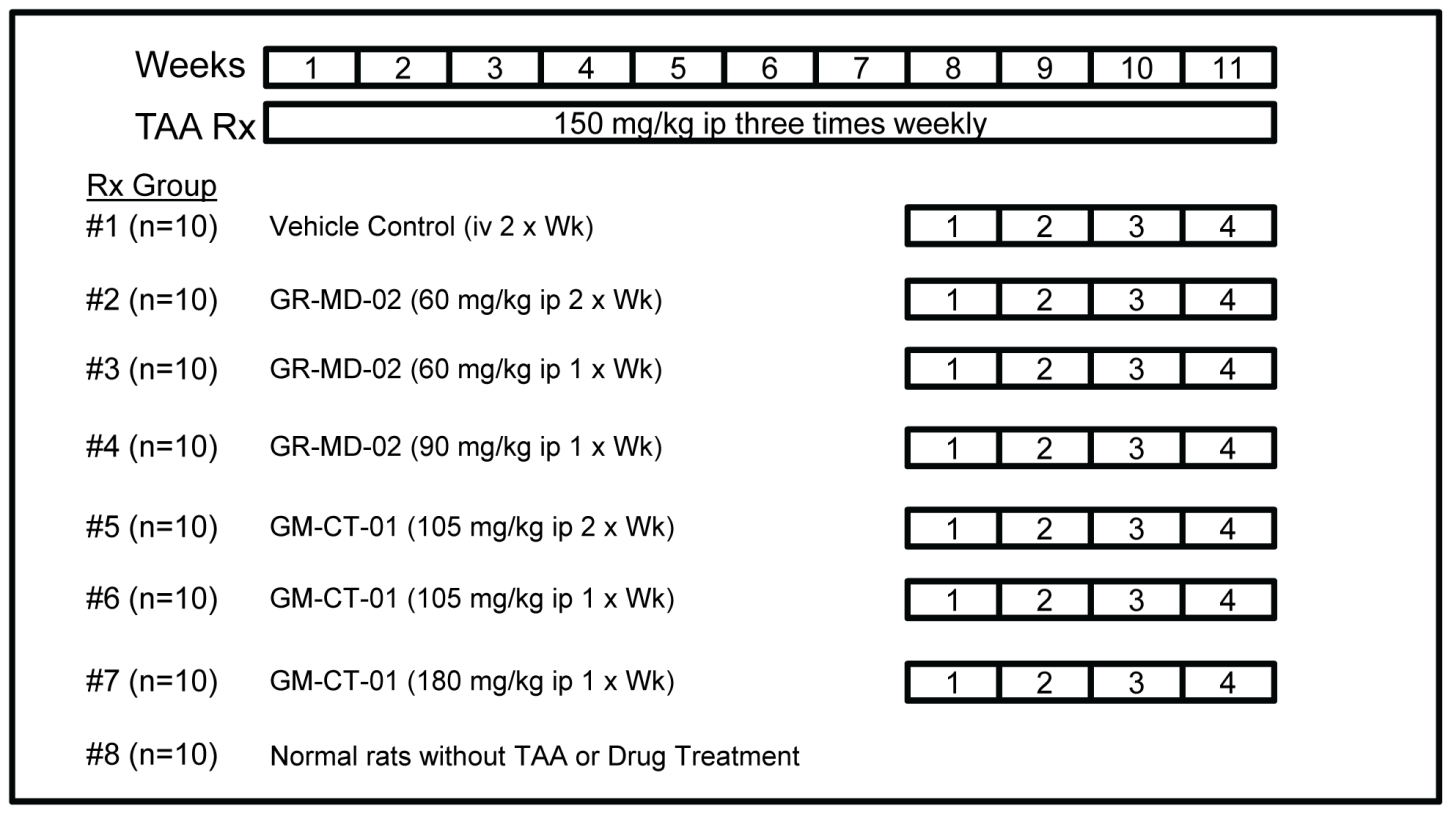
Experimental design. TAA = thioacetamide; ip = intraperitoneal injection.

In this experiment, treatment with GR-MD-02 was compared with GM-CT-01 in a more advanced stage of fibrosis induced by more intense regimen of TAA administration. Rats were treated with TAA for eleven weeks, for a cumulative dose of TAA of 4950 mg/kg, and in the final four weeks GR-MD-02 or GM-CT-01 treatment was added using various dosing schedules (Figure 4). A key feature of this experimental design was that TAA treatment was continued during administration of drug treatments. Animals tolerated the treatments well with no obvious adverse effects observed.

**Figure 5:**
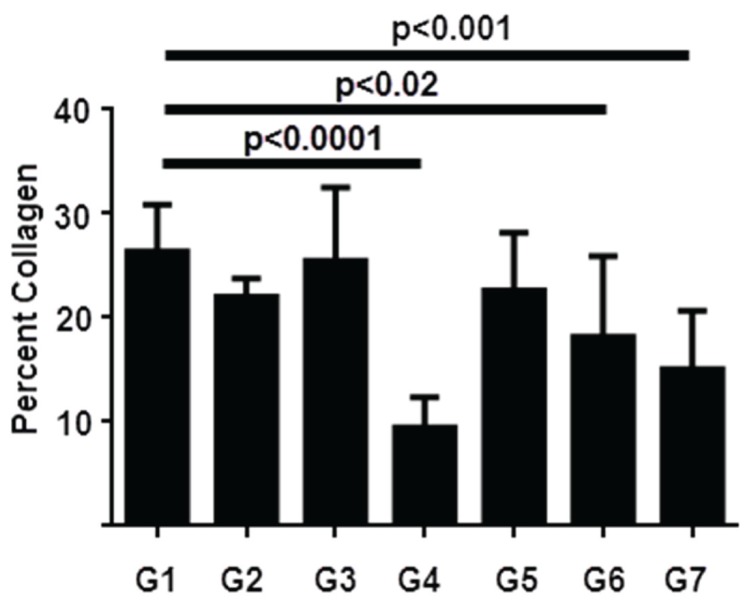
Graphical representation of the percentage Sirius red positive tissue from experiment described in **Figure 4**. Statistical analysis performed was One Way ANOVA followed by Dunnett’s multiple comparison testing to compare each group separately to group 1. Mean values, standard deviation, and adjusted p values are shown.

**Figure 6:**
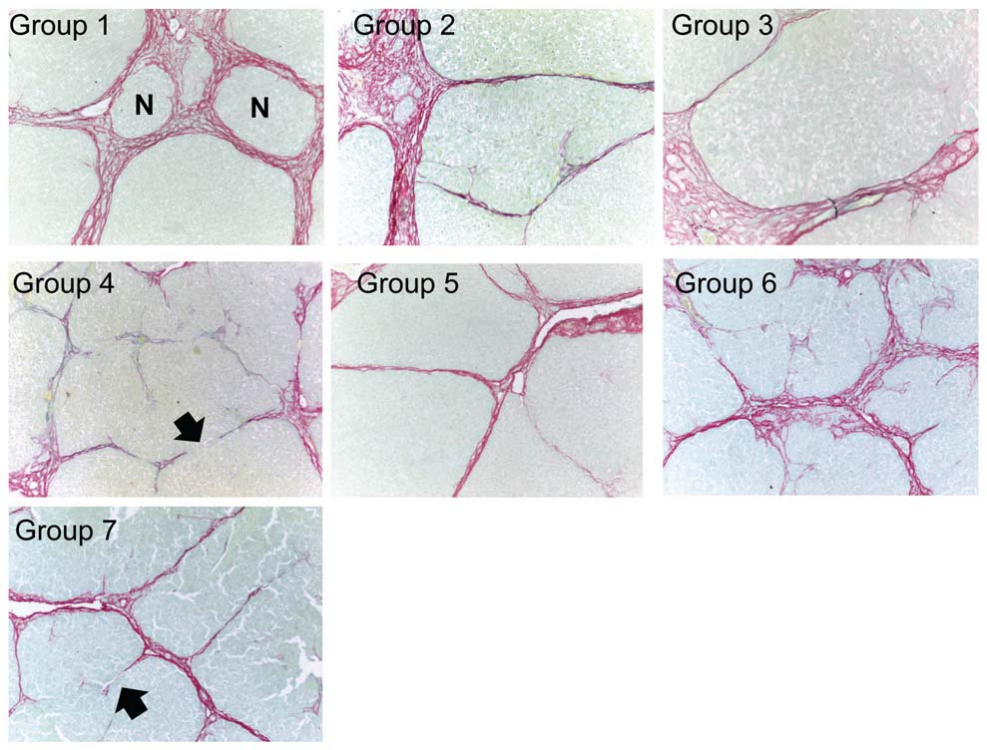
Representative histology of Sirius red stained liver sections from experiment described in **Figure 4**. A photomicrograph was chosen from each experimental group that was approximately equal to the mean of the group for percent area stained with Sirius red. N = nodule; Closed arrows = incomplete (broken) strands of bridging fibrosis.

Figure 5 depicts a graphical representation of Sirius red staining and Figure 6 shows representative histological pictures of each group chosen to approximate the mean of the quantitative analysis. Rats in this experiment developed a robust degree of fibrosis, with an average of over 25% of the liver staining with Sirius red in the vehicle-treated control animals. Both GR-MD-02 and GM-CT-01 treatment reduced fibrosis. GR-MD-02 at 60 mg/kg twice weekly showed a small, not statistically significant, reduction in Sirius red staining, whereas there was no effect when 60 mg/kg was given once weekly. In contrast, GR-MD-02 at a dose of 90 mg/kg given once weekly markedly reduced Sirius red staining by over 2.5 fold in comparison to vehicle treated control rats, which was highly statistically significant. A very similar pattern was seen with GM-CT-01 with the greatest effect seen with treatment of 180 mg/kg given once weekly.

**Figure 7:**
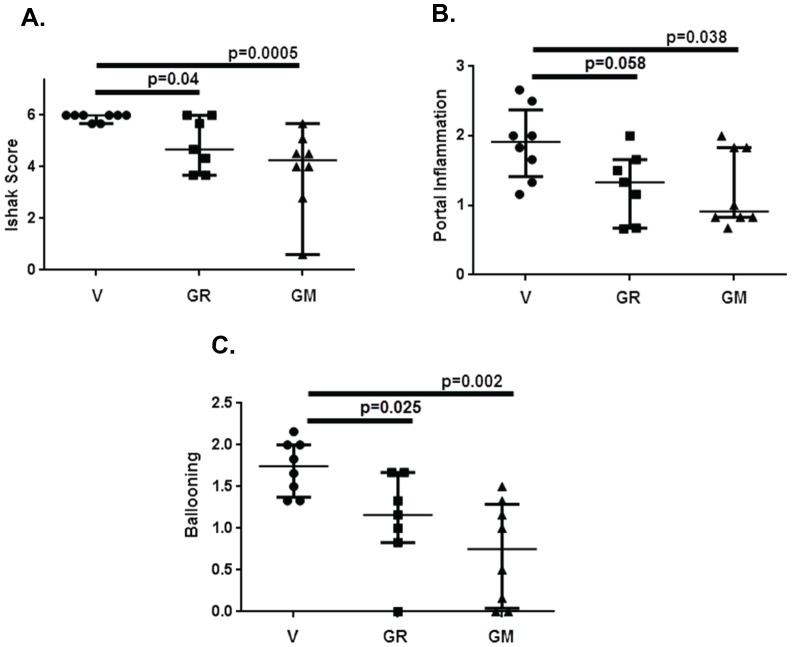
Histological analysis of liver sections from groups 1(V), 4 (GR) and 7 (GM) from experiment described in **Figure 4**. Liver histology from each animal was evaluated in a blinded fashion by an experienced pathologist as described in Materials and Methods. Statistical analysis was done using Mann-Whitney test for non-parametric measurements and the graph show median with interquartile range. **A**: Ishak Score; B: Portal Inflammation; C: Ballooning degeneration of hepatocytes.

Histological staging was performed on 6 independent slides from each animal in Groups 1, 4 and 7 by an experienced liver pathologist who was blinded to the treatment conditions. This analysis showed that all of the vehicle treated control animals had Ishak stage 6 fibrosis, or cirrhosis (Figure 7A), with thick bands of collagen and nodule formation (Figure 6). Liver sections from animals treated with GR-MD-02 and GM-CT-01 had a significant reduction in the stage of fibrosis with most showing reversal of cirrhosis (Figure 7A). Representative slides in Figure 6 show markedly thinned fibrous septae with obvious incomplete septa, highlighted with arrows. These findings indicate that, in the presence of continued TAA treatment, both GR-MD-02 and GM-CT-01 promoted regression of collagen and reversal of the architectural changes associated with cirrhosis.

Portal inflammation and ballooning degeneration of hepatocytes was reduced in animals treated with GR-MD-02 and GM-CT-01 when compared with vehicle controls. The analysis of all animals for portal inflammation showed significant reduction for GM-CT-01 and near significance for GR-MD-02 (Figure 7B). Ballooning degeneration of hepatocytes was significantly reduced in both treatment groups when compared control (Figure 7C). There was evidence of ductal reactivity in vehicle treated control (mean score 2.14), but there was no significant reduction in treated animals. However, there was more ductal atypia in control animals (6 of 8) than in GR-MD-02 (2 of 7) and GM-CT-01 (3 of 8) treated animals. There was minimal lobular inflammation and steatosis in all animals.

Portal pressure measurements of normal rats were compared to those of the experimental groups (Table 1). The vehicle-treated control animals had a markedly elevated portal pressure consistent with cirrhosis as evident by histopathology. Two of the treated groups, both administered GR-MD-02, had a statistically significant reduction in portal pressure, and several other groups had pressures that trended lower. These data demonstrate that the reduction of liver fibrosis and reversal of architectural changes of cirrhosis were also associated with an improvement in portal hypertension.

**Table 1:** Portal pressures in rats at sacrifice.

Treatments	Portal Pressure (cm of water)
	Mean (Standard Deviation)
Group 1: Vehicle Control	20.3 (2.4)
Group 2: GR-MD-02	15.7 (2.9)*
Group 3: GR-MD-02	18.9 (1.4)
Group 4: GR-MD-02	17.1 (2.4)*
Group 5: GM-CT-01	20.8 (1.9)
Group 6: GM-CT-01	19.6 (2.4)
Group 7: GM-CT-01	18.5 (3.7)
Normal Rats	10.5 (2.4)**

P values compared to vehicle control (control 0.9% NaCl).

* p<0.05;

** p<0.001.

Serum transaminases were elevated in fibrotic rats (Table 2) and several groups had reductions compared to the vehicle-treated control group, but these measurements did not clearly parallel the effects on the measurements of fibrosis.

**Table 2:** Biochemical data from experiment shown in Figure 4.

Treatment	% Collagen(Sirius Red)	TGFβ-R1(mRNA)	Collagen(mRNA)	Collagen(protein)	α-SMA(mRNA)	α-SMA(protein)	AST(IU/L)	ALT(IU/L)
Group 1 (Vehicle)	26 (4.5)	1 (0.06)	1	1 (0.3)	1	1 (0.25)	121 (27)	107 (25)
Group 2 (GR)	22 (1.8)*	1.19 (0.08)	1 (0.37)	0.45 (0.05)	0.81 (0.27)	0.7 (0.41)	112 (58)	97 (16)
Group 3 (GR)	25.5 (7)	0.81 (0.22)	0.69 (0.12)*	0.68 (0.22)	0.77 (0.46)	0.99 (0.14)	86 (20)*	88 (16)
Group 4 (GR)	9.5 (2.9)**	1.06 (0.56)	0.77 (0.18)*	0.29 (0.06)*	0.58 (0.19)*	0.67 (0.17)	115 (30)	93 (17)
Group 5 (GM)	23.5 (5.5)	1.14 (0.17)	0.93 (0.33)	0.83 (0.4)	0.96 (0.19)	1.2 (0.28)	108 (19)	93 (17)
Group 6 (GM)	18 (7.7)*	1.19 (0.42)	1.1 (0.26)	0.35 (0.13)*	0.92 (0.38)	0.65 (0.1)	91 (11)*	82 (12)*
Group 7 (GM)	15 (5.6)**	1.67 (0.44)	0.77 (0.26)	0.23(0.17)*	0.88 (0.42)	0.47 (0.24)*	82 (15)*	77 (10)*
Normal		0.28	0.2	0.06 (0.01)**	0.35	0.14 (0.01)*	80 (11)*	70 (8)*

% Collagen is percent area of biopsy stained with Sirius red. TGFb-R1, Collagen, and a-SMA mRNA were assessed by RT PCR and Collagen and α-SMA protein assessed by Western Blot (protein for TGFb-R1 was not evaluated). Data is normalized to Group 1 (Vehicle). Data is presented as mean and standard deviation in parentheses. Two sided t-text evaluated for Groups 2–7 as compared to Group 1 (Vehicle).

* p<0.05;

** p<0.001.

### Evaluation of Galectin-3 Expression

**Figure 8:**
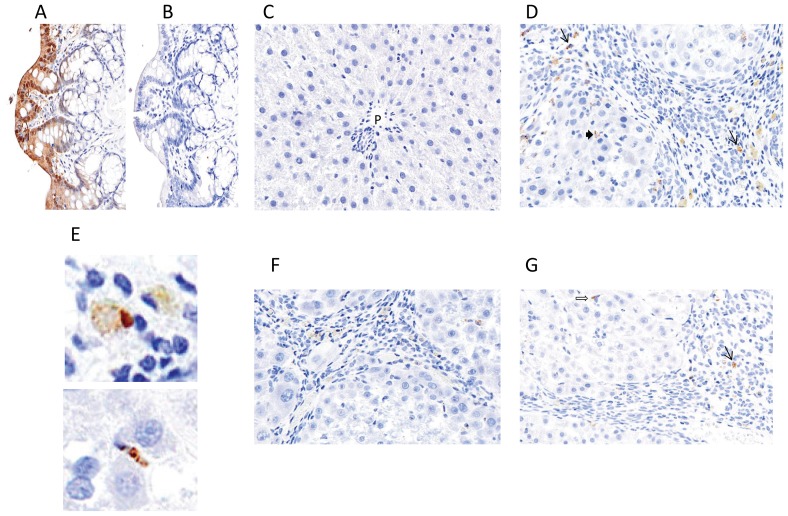
Galectin-3 Immunohistochemsitry. A: Rat colon B. Rat colon with omission of primary galectin-3 antibody; C: Normal rat liver; D: Rat liver treated with TAA as in figure 4 and treated with vehicle (Group 1); E: upper panel shows high power of stained portal macrophages; lower panel shows stained lobular sinusoidal cell with morphology of a stellate cell. F: Rat liver treated with TAA as in figure 4 and treated with GR-MD-02 (Group 4); G: Rat liver treated with TAA as in figure 4 and treated with GM-CT-01 (Group 7).

Galectin-3 protein expression was evaluated using immunohistochemistry. For a positive control for galectin-3 staining, rat colon demonstrated strong staining of colonic epithelium (Figure 8A) and there was no non-specific staining when the primary antibody was eliminated from the staining protocol (Figure 8B). Normal liver sections had very little staining with only scattered and rare staining of Kupffer cells (Figure 8C), as previously described in the literature [14]. TAA-treated liver from vehicle control group (Group 1) showed galectin-3 staining in both liver lobules and prominently in expanded portal tracts and fibrous septa (Figure 8D). The cells staining in portal areas and fibrous septa had the morphology of macrophages, as shown in higher magnification in the upper panel of Figure 8E. In the liver lobules there was staining of cells that appeared to be Kupffer cells (Figure 8D) as well as elongated sinusoidal cells that had the appearance of stellate cells (lower panel of Figure 8E). The predominant staining of portal tracts and fibrous septa in fibrotic animals treated with TAA is consistent with what has been previously described in the literature for both rats treated with TAA [14] and mice treated with carbon tetrachloride [8].

The pattern of staining in animals treated with either GR-MD-02 (Group 4) or GM-CT-01 (Group 7) was similar in both groups and is shown in Figures 8F and 8G, respectively. There was a reduction in the number of portal and septal macrophages that stained with galectin-3 in comparison to the numbers in the vehicle control group, and those cells that did stain in Group 4 appeared to have more pale staining. There was staining of scattered lobular cells that appeared to Kupffer and stellate cells in both groups which was not demonstrably different than the staining in the vehicle control group. These results suggest that there is a marked increase in cells that express galectin-3 in rats with TAA-induced fibrosis and that treatment with both drugs resulted in a decreased number of galectin-3 expressing macrophages in portal areas and fibrous septa.

### Molecular Analysis of TAA-induced Liver Fibrosis

A number of mRNAs from genes driving the fibrotic process were assessed by real time PCR in the livers of rats from the experiment comparing GR-MD-02 and GM-CT-01 including, collagen 1 (COL1), alpha-1 smooth muscle actin (α-SMA), beta platelet derived growth factor receptor (β-PDGFR), transforming growth factorβ receptor-1 (TGFBR1), matrix metalloproteinase 1 and 2 (MMP-1 and MMP-2), and tissue inhibitor of metalloproteinase 1 and 2 (TIMP-1 and TIMP-2). Three of these markers were compared to normal liver (Table 2). The expression of TGFBR1 mRNA was increased in vehicle- and TAA-treated rat liver by 3.6-fold compared to normal rat liver (Table 2). Likewise, the expression of α-SMA mRNA was increased 2.8-fold in vehicle-treated animals and COL1 mRNA was increased 5-fold (Table 2).

Fibrosis-associated mRNA expression in treated animals was compared to the vehicle-treated control group. In comparison to the vehicle-treated control group, the levels COL1 mRNA were reduced by 31% (p<0.05) and 23% (p<0.05) in groups 3 and 4 treated with GR-MD-02, respectively. There was a trend towards reduced COL1 mRNA in group 7 treated with GM-CT-01, but this did not reach significance (Table 2). The expression of α-SMA mRNA trended lower in a number of groups, but only reached significance in group 4 (GR-MD-02) with a 42% reduction (p<0.05) (Table 2). TGFβ-R1 mRNA was not significantly different between vehicle and treated groups (Table 2). The analysis of other transcripts did not show significant differences from the vehicle control animals, including βPDGF-R, TIMP-1, TIMP-2 and MMP-2 (data not shown).

**Figure 9:**
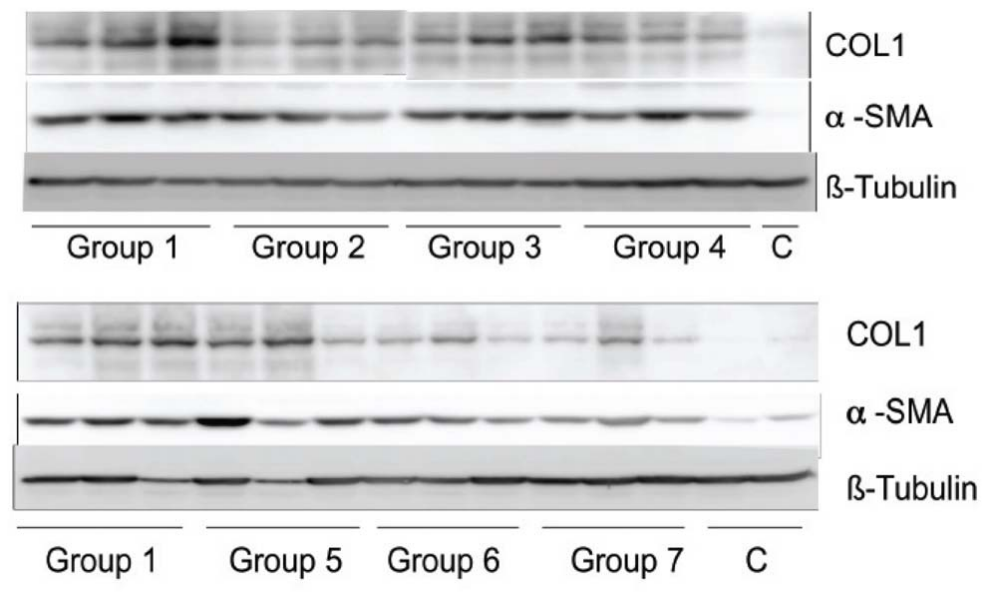
Western blot analysis of protein isolated from livers from animals in the experiment described in **Figure 4**. COL1 = collagen type 1; α-SMA = alpha smooth muscle actin; C = normal rat liver protein; β-tubulin was used as an internal control.

Western blot was performed to evaluate differences in COL1 and α-SMA protein levels (Figure 9). For collagen protein there was a trend towards decreased expression in all treated groups compared to vehicle-treated animals, with statistically significantly reduced levels in group 4 treated with GR-MD-02 and groups 6 and 7 treated with GM-CT-01 (Table 2). The levels of α-SMA protein was somewhat more variable, but did show a trend to a reduction in animals group 4 treated with GR-MD-02 and statistically significant reduction in group 7 animals treated with GM-CT-01 (Table 2). Of note, the reduction in collagen protein was greater than the relative degree of reduction in COL1 mRNA. Protein levels associated with the other mRNAs were not evaluated.

### Minimal Direct Effects of Galectin-3 Inhibitors on Cultured Stellate Cells

To begin exploring potential mechanisms underlying the anti-fibrotic efficacy of the galectin-3 inhibitors, we employed the LX-2 cell line which is an immortalized human hepatic stellate cell line has been used extensively to evaluate mechanisms of activation and expression of fibrogenic mediators [2], [12], [15]. Immunohistochemistry confirmed that this cell line expressed both galectin-1 and galectin-3 (data not shown). This cell line was used to evaluate the effect of the anti-galectin complex carbohydrates.

**Figure 10:**
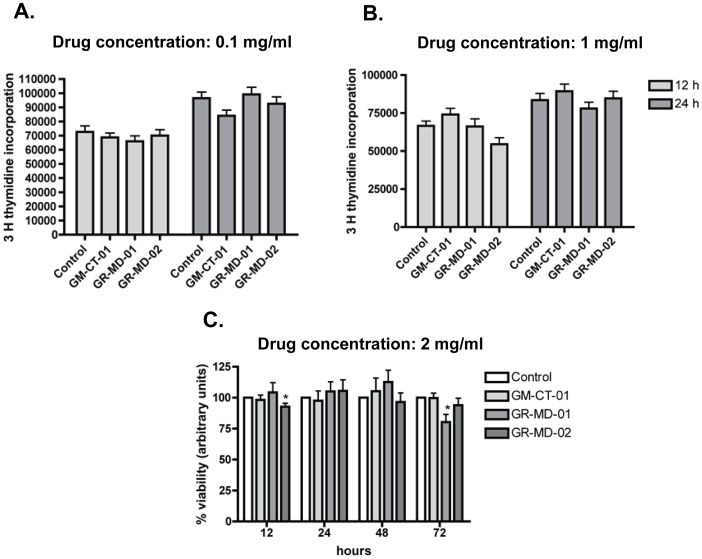
Evaluation of compound effects on growth and viability of LX-2 cells. **A**: thymidine incorporation at 12 and 24 hours of culture with drug compounds at a concentration of 0.1 mg/ml. **B**: thymidine incorporation at 12 and 24 hours of culture with drug compounds at a concentration of 1.0 mg/ml. **C**: cellular viability at 12, 24, 48, 72 hours of culture with drug compounds at a concentration of 2 mg/ml.

**Figure 11:**
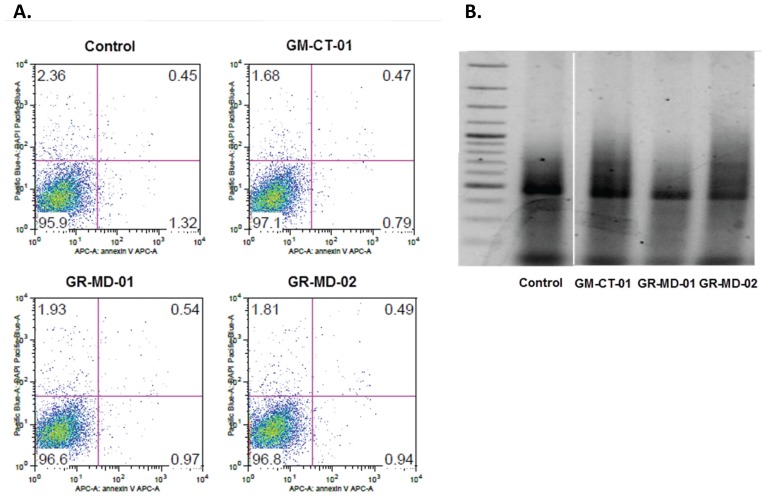
Evaluation of LX-2 cells for apoptosis. A. Fluorescent activated cell sorting following treatment with Annexin V apoptosis detection kit APC (Ebioscience). B. 2% agarose gel, stained with ethidium bromide, and visualized by transillumination with UV light after using Apoptotic DNA Ladder Extraction Kit (BioVision, Mountain View. Samples were all analyzed on same gel; discontinuity on the figure is due to the removal of repeated samples of different lots of GM-CT-01 which gave same results.

LX-2 cells were treated with incremental concentrations of the anti-galectin drugs. Increasing concentrations of each drug used in these studies did not affect cell growth or viability (Figure 10). Moreover, there was no evidence of apoptosis using the annexin V apoptosis detection kit APC (Ebioscience) or by examination of DNA fragmentation (Figure 11).

**Figure 12:**
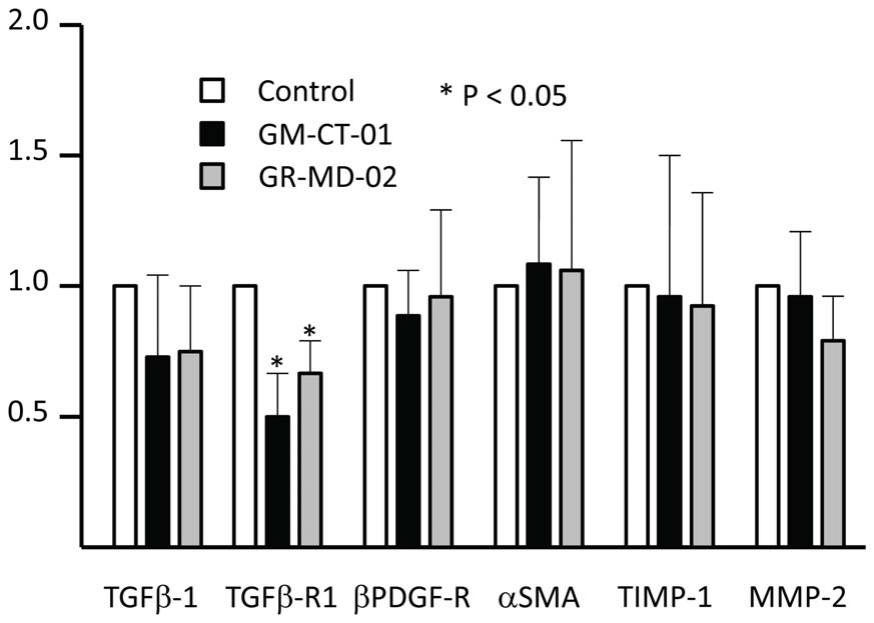
mRNA expression in LX-2 cells after 48 hours of culture. Drug concentrations were 0.1/ml culture media. Data are expressed as mean and standard deviation. Statistics performed with t-tests as compared to control.

**Figure 13:**
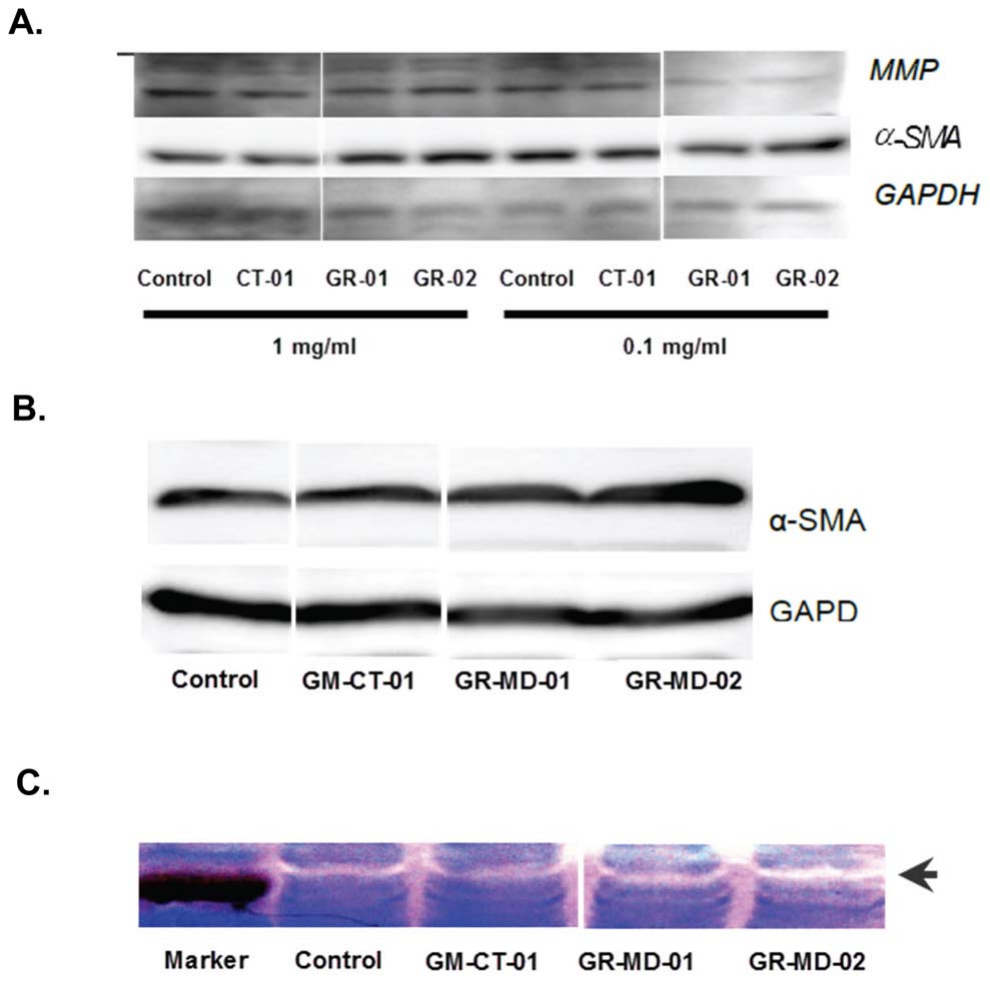
Protein expression and gelatinase activity in stellate cell cultures. Samples were all analyzed on same gel or autoradiograph; discontinuity on the figures is due to the removal of repeated samples of different lots of GM-CT-01 which gave same results. **A**: Western blot analysis of protein isolated from cultures of LX-2 cells. MMP2 = metal metalloprotease 2; α-SMA = alpha smooth muscle actin; GAPDH was used as an internal control. CT-01 = GM-CT-01; MD-01 = GR-MD-01; MD-02 = GR-MD-02. **B**: Western blot analysis of protein isolated from cultures of primary human stellate cells. α-SMA = alpha smooth muscle actin; GAPDH was used as an internal control. All drug concentrations were 1 mg/ml and evaluations were performed 24 hours following addition of drug. **C**: MMP2 activity by zymography. MMP-2 enzymatic activity in LX-2 was determined using 10% Zymogram (Gelatin) gel (10% Tris-Glycine gel with 0.1% gelatin substrate). Gelatinases present in the cells degrade the gelatin matrix, leaving a clear band (arrow) after staining the gel for protein. Drugs were administered to cells at 0.1 mg/ml for 24 hours.

mRNAs associated with fibrogenesis and stellate cell activation were assessed in LX-2 following treatment with GR-MD-02 and GM-CT-01 including, α-SMA, β-PDGFR, TGF-β1, TGFBR1, MMP-2, and TIMP-1. Following culture for 12 and 24 hours there no significant changes in any of these transcripts. However, there was a significant decrease in TGFBR1 mRNA at 48 hours following treatment with both drugs (Figure 12). These findings were corroborated by protein analysis for MMP2 and α-SMA where there was no change with treatment of increasing doses of the drugs (Figure 13A). The zymography results showed that metalloprotease enzyme activity was high in LX-2 cells and there was no change with drug treatment (Figure 13B). Primary cultures of human stellate cells were also evaluated and, like the LX-2 cells, there was no change in α-SMA protein with drug treatment (Figure 13B).

The results of these cell culture experiments show that there were no dramatic effects on stellate cells that would adequately explain the effects seen in the in vivo experiments. However, the changes in the TGF-β receptor-1 might reduce the ability of TGF-β to activate stellate cells.

## Discussion

Our findings indicate that the two agents, GR-MD-02 and GM-CT-01 have a marked therapeutic effect on the histology of liver fibrosis induced by thioacetamide treatment in rats. In addition to a reduction in collagen content, these agents reduced the bridging fibrosis and histological cirrhosis despite continued exposure to thioacetamide. Moreover, there was a significant reduction in portal hypertension. The vehicle-treated cirrhotic rats had a doubling of the portal pressure compared to normal rats, whereas those in the treated groups, particularly with GR-MD-02, had a significant reduction in portal pressure. Therefore, it appears that treatment with these agents not only leads to degradation of collagen and regression of histological findings of advanced fibrosis and cirrhosis, but also attenuates the pathophysiologic consequences of cirrhosis.

The complex carbohydrate drugs were chosen based on the hypothesis that inhibition of galectin molecules, and specifically galectin-3, might prove to be a therapy for liver fibrosis. Mice that lack the galectin-3 gene are viable, fertile, generally healthy and have a normal life span [16], but they do have a late inflammatory response to acute peritonitis [17]. Galectin-3 null mice have markedly reduced collagen accumulation in the liver following treatment with carbon tetrachloride [8]. In these studies the lack of galectin-3 appeared to be linked to reduced activation of hepatic stellate cells in the liver. Based on these and other studies in kidney [11], [18], lung [10], and heart [19], galectin-3 appears to be integral to accumulation of fibrosis in parenchymal tissue.

While the mechanism for these effects on fibrosis and cirrhosis is presumed to be interaction of the drugs with the galectin-3 protein, the interactions with galectin molecules are complex and molecular events downstream of galectin proteins are poorly understood. Galectin-3 has a carbohydrate binding domain (CRD) which is shared among galectin proteins [5], but in contrast to other galectin proteins, it has a long N-terminal domain that is involved in forming multimers [20]. Galectin-3 binds poorly to single galactose molecules [21], more avidly to galactose containing disaccharides [22], and most avidly to larger molecules such as glycoproteins with terminal galactose residues [21]. Although galectins are defined by their ability to bind to model carbohydrates containing galactose, such as lactosamine, the individual galectins appear to bind to different sets of glycans on glycoproteins, thus providing specificity between galectins [23]. For example, galectin-1 and galectin-3 bind to distinct cell surface receptors on T-cells [24]. There are many potential ligands for the lectin properties of galectin-3 including laminin, integrins, collagens, fibronectin, elastin, mucins, CD4+, CD8+, TGFBR, neural cell adhesion molecules, and many others [25]. Binding of galectin-3 to N-glycans has been connected to multiple cellular processes including cell adhesion and migration, immune cell function, inflammation, and neoplasia [5], [26]–[30]. It is likely, that inhibition of galectin-3 modulates multiple protein interactions in the extracellular space thereby altering cellular function. In addition to glycan interactions, there are protein-protein interactions that occur with un-glycosylated proteins, mainly in the nucleus and cytoplasm [31]. It appears, therefore, that the extracellular effects of galectins are related to their lectin properties to bind to glycoproteins whereas their intracellular effects are more related to protein-protein interactions.

GR-MD-02 and GM-CT-01 are complex carbohydrate molecules which present N-terminal galactose residues that are capable of interacting with galectin. Heteronuclear single quantum coherence (HSQC) nuclear magnetic resonance (NMR) spectroscopy was used to confirm the association of these complex carbohydrates with various domains of galectin proteins [32]–[35]. The domains on galectin proteins that bind to these complex carbohydrates were found to be more complicated than the binding for disaccharide and oligosaccharides [21], or compounds based on disaccharides that have a higher affinity for the CRD [36]. Mapping of the binding sites on galectin-1 that interact with GM-CT-01 showed the most intense binding was to the F-face of the molecule which traverses the protein dimerization domain, with minimal interaction at the canonical CRD (S-face) [32]. Similarly, while GR-MD compounds bind to the CRD, they also bind to a larger region on the protein than small saccharides interacting with both S-face and F-face of galectin-1 [37]. Additionally, both complex carbohydrates bind to multiple molecules of galectin per molecule of carbohydrate. We have also shown that both GM and GR carbohydrates bind to the galectin-3 CRD through somewhat different sets of amino acid residues and the affinity at 50% saturation of GR-MD-02 and GM-CT-01 to galectin-3 is 2.9 µM and 2.8 µM, respectively (unpublished data). This compares to previously published data on galectin-1 binding affinities for GR-MD-02 and GM-CT-01 of 8 µM and 10 µM, respectively [32], [33], [37]. The high molecular weight of these compounds and the lectin binding properties suggest that they likely act predominantly on extracellular galectins.

The potential mechanisms by which these galectin-3 binding drugs might have the demonstrated effect on fibrosis are not yet clear. Henderson, et al. showed that galectin-3 appeared to be required for activation of hepatic stellate cells to myofibroblasts [8]. A reduction in activated stellate cells would clearly be important as they represent the primary cell for synthesis of extracellular collagen in liver fibrosis [1], [2], [15], [38], [39]. In our experiments, there was a decrease in α-SMA protein in those treatment groups with the greatest anti-fibrotic effect, consistent with a reduction in stellate cell activation in response to the therapy. However this does not necessarily reflect a direct effect on stellate cells rather than an indirect effect by modulating the nature or extent of inflammation. Indeed, the effect of the drugs on isolated stellate cells and the LX-2 stellate cell line was extremely modest and therefore unlikely to account entirely for the significant efficacy in vivo.

Future studies will need to determine whether the primary effect of these compounds in liver is via inhibition of galectin-3 on stellate cells, or through an indirect effect of changes in the cytokine and/or inflammatory milieu. Prior studies assessed the effect of galectin-1 and galectin-3 on proliferation and activation of cultured stellate cells [40], [41] and the effect of galectin-3 on phagocytosis-dependent activation of stellate cells [42]. In our experiments there was no effect in LX-2 cells on proliferation, apoptosis, or the expression of most fibrogenesis-related genes and proteins. There was a reduction in the expression of TGF-β receptor-1 gene expression following treatment with both GR-MD-02 and GM-CT-01. TGF-β is an important cytokine in fibrogenesis and for the activation of stellate cells. Additionally, there is evidence that the activity of the TGF-β receptor in lung fibrosis is dependent on galectin-3 protein and that inhibition of galectin-3 is inhibits receptor activity [10]. Therefore, inhibition of TGF-β-dependent stellate cell activation may be one mechanism that drug inhibition of galectin-3 could provide some of the effect seen in this animal model.

The macrophage is another potential target by which galectin-3 binding drugs might affect fibrosis. Macrophages are pivotal to the development and resolution of collagen deposition in organs [43] and are clearly important in liver fibrosis [44]. Moreover, it is now clear that activated macrophages differentiate into a number of different subtypes, referred to as macrophage polarization, which have distinct functions along the continuum from inflammation and fibrogenesis to resolution of fibrosis. The classically activated M1-macrophages have an acute inflammatory phenotype, are aggressively phagocytic for bacteria, and produce large amounts of cytokines. The alternatively activated, anti-inflammatory M2-macrophages can be separated into three subgroups that have different function in immune regulation, tolerance, and tissue repair or wound healing. Recently, a new subtype of M2-macrophages was identified that is critical for resolution of fibrosis in the liver [45].

While expressed in many immune and other cell types, galectin-3 was first described in macrophages as Mac-2 and is expressed at much higher levels in macrophages than other cell types [46]. In addition, several lines of evidence suggest that galectin-3 is important for macrophage function in fibrotic disease [6], [11], [18], including regulation of alternative activation of macrophages [18]. In the experiments described, the regression of cirrhosis and fibrosis in a short time frame with continued toxin treatment and the presence of incomplete septa suggest that there is a relatively rapid degradation of collagen. Macrophages located in portal tracts and fibrotic areas were the predominant cell type that expressed galectin-3 in immunohistochemistry of cirrhotic livers in this study. Moreover, drug treatment reduced the number of macrophages expressing galectin-3. These data suggest that macrophages may be a primary target for these drug compounds. Future studies will assess whether interaction with galectin-3 by these compounds alters macrophage polarization, thereby reducing pro-inflammatory macrophages and increasing reparative macrophages that can degrade collagen.

In summary, we have demonstrated that galectin-binding, complex carbohydrate drugs can provoke regression of fibrosis and histological changes of cirrhosis in a toxin-induced model of liver fibrosis in the rat. Moreover, the regression in cirrhosis is associated with a reduction in portal hypertension, demonstrating that the change in liver architecture has a physiological effect on liver blood flow and/or resistance. These findings suggest that treatment with complex carbohydrate drugs that bind galectin-3 may represent a therapeutic approach that may be useful in the therapy of advanced fibrosis and cirrhosis in humans, especially as they appear to be extremely well tolerated.
